# X4 Human Immunodeficiency Virus Type 1 gp120 Promotes Human Hepatic Stellate Cell Activation and Collagen I Expression through Interactions with CXCR4

**DOI:** 10.1371/journal.pone.0033659

**Published:** 2012-03-27

**Authors:** Feng Hong, Yedidya Saiman, Chuanping Si, Arevik Mosoian, Meena B. Bansal

**Affiliations:** 1 Department of Medicine, Mount Sinai School of Medicine, New York, New York, United States of America; 2 Department of Immunology, Jining Medical College, Jining, China; University of Nebraska Medical Center, United States of America

## Abstract

**Background & Aims:**

Patients coinfected with HIV-1 and HCV develop more rapid liver fibrosis than patients monoinfected with HCV. HIV RNA levels correlate with fibrosis progression implicating HIV directly in the fibrotic process. While activated hepatic stellate cells (HSCs) express the 2 major HIV chemokine coreceptors, CXCR4 and CCR5, little is known about the pro-fibrogenic effects of the HIV-1 envelope protein, gp120, on HSCs. We therefore examined the *in vitro* impact of X4 gp120 on HSC activation, collagen I expression, and underlying signaling pathways and examined the *in vivo* expression of gp120 in HIV/HCV coinfected livers.

**Methods:**

Primary human HSCs and LX-2 cells, a human HSC line, were challenged with X4 gp120 and expression of fibrogenic markers assessed by qRT-PCR and Western blot +/− either CXCR4-targeted shRNA or anti-CXCR4 neutralizing antibody. Downstream intracellular signaling pathways were evaluated with Western blot and pre-treatment with specific pathway inhibitors. Gp120 immunostaining was performed on HIV/HCV coinfected liver biopsies.

**Results:**

X4 gp 120 significantly increased expression of alpha-smooth muscle actin (a-SMA) and collagen I in HSCs which was blocked by pre-incubation with either CXCR4-targeted shRNA or anti-CXCR4 neutralizing antibody. Furthermore, X4 gp120 promoted Extracellular signal-regulated kinase (ERK) 1/2 phosphorylation and pretreatment with an ERK inhibitor attenuated HSC activation and collagen I expression. Sinusoidal staining for gp120 was evident in HIV/HCV coinfected livers.

**Conclusions:**

X4 HIV-1 gp120 is pro-fibrogenic through its interactions with CXCR4 on activated HSCs. The availability of small molecule inhibitors to CXCR4 make this a potential anti-fibrotic target in HIV/HCV coinfected patients.

## Introduction

HIV prevalence in the US is increasing due to a combination of the stable incidence of HIV (estimated at 53,600 new cases per year in 2006) and longer life expectancy due to effective antiretroviral therapies (ART) [1]. As HIV patients continue to live longer in the setting of effective ART, liver disease has become the leading cause of non-AIDS related mortality [2]. Due to shared routes of transmission, HCV and HBV are common in HIV-infected patients though ethanol-induced liver disease is also prevalent. Approximately 300,000 individuals in the US are coinfected with HCV and HIV [3], [4]. Median time to cirrhosis in HIV/HCV coinfected patients is approximately 12 years shorter than HCV monoinfected patients [5], [6]. While immune dysregulation in the setting of HIV infection may play a role in accelerating liver fibrosis from HCV, recent studies suggest faster fibrosis progression in HIV/HCV coinfected patients despite effective anti-retroviral therapy [7]. Furthermore, while separating the impact of decreased CD4 count from HIV RNA levels is difficult, cohort studies suggest independent effects of HIV RNA on fibrosis progression [1], [8], [9]. In one study, a dose-dependent effect of HIV RNA levels on fibrosis progression rates was observed, further implicating the virus in enhanced liver fibrogenesis [8].

The liver is unique in that the majority of its blood flow is derived from the portal circulation. As blood enters the liver, it is distributed through the hepatic sinusoids, which are lined by a uniquely fenestrated endothelium. HSCs reside between the fenestrated endothelial cells and hepatocytes. Consequently, the low pressure flow combined with the fenestrations within the sinusoids create an environment that is primed for interactions between gut-derived pathogens, intrahepatic cell populations, and circulating cells of the immune system. Hepatic fibrosis is a wound-healing process that occurs when the liver is chronically injured. A central mediator of this fibrotic process is the activated HSC [10]. With liver injury, this normally quiescent cell is transformed into a myofibroblastic cell that is fibrogenic, proliferative, and contractile [10]. This transformation process or “activation” is believed to occur in 2 phases: Initiation and Perpetuation. Initiation occurs in response to factors such as oxidative stress, hepatocellular injury/apoptosis, and LPS [11]. Once activated, the HSC becomes responsive to paracrine stimuli such as transforming growth factor-beta 1 (TGF- ß1) [12] and a number of autocrine pathways are set in motion [13]. This process is dynamic and once the HSC is “activated”, additional factors/stimuli can further accentuate features of activation such as production of collagen I and perpetuate this phenotype.

The chemokine receptors, CCR5 and CXCR4, are co-receptors for R5-tropic and X4-tropic HIV-1, respectively [14]. The HIV envelope protein, gp120, from R5-tropic HIV-1 binds CCR5 while gp120 from X4-tropic HIV-1 binds CXCR4. Gp120 is capable of eliciting biologic effects in target cells even in the absence of true infection [15]. While numerous studies have examined the effects of gp120 on target cells, little in known about the effects of gp120 on intrahepatic cell populations. X4 HIV-1 gp120 promotes hepatocyte apoptosis [16] and both X4 and R5 gp120 act cooperatively with the HCV E2 glycoprotein to promote hepatocyte apoptosis and secretion of the pro-inflammatory cytokine, IL-8 *in vitro* [17]. More recently, it was shown that both X4 and R5 gp120 promote TGF-ß1 production by HCV replicon cells through interactions with CXCR4 and CCR5 [18]. While both hepatocyte apoptosis and TGFß1 promote HSC activation and fibrogenesis, little is known about the direct effects of gp120 on activated HSCs.

The direct effect of viral proteins on HSCs has been previously demonstrated. Both HCV [19], [20], [21] and HBV proteins [22] modulate the biology of activated HSCs potentially impacting on the progression of liver fibrosis. Activated HSCs express both HIV-1 chemokine coreceptors, CXCR4 and CCR5 [23], [24] and we have already established that expression of CXCR4 is increased in HCV-infected livers [24] and increases with culture-induced HSC activation [24]. Moreover, stromal cell-derived factor-1 alpha (SDF-1*a*), the endogenous ligand for CXCR4, promotes HSC collagen I expression, activation, and proliferation [24]. We have also shown that HIV infection of activated HSCs *in vitro* is associated with upregulation of collagen I and the pro-inflammatory chemokine, monocyte chemoattractant protein (MCP-1) [25]. It is not clear whether these effects require infection or result from extracellular gp120 interactions with CXCR4 and CCR5. Bruno *et al.* demonstrated that primarily R5 gp120 promoted MCP-1 and IL-6 secretion, TIMP-1 expression, and the directional migration of HSCs [26] suggesting that HIV envelope protein can elicit pro-inflammatory and to a lesser extent pro-fibrogenic effects in activated HSCs through interactions with CCR5.

HIV-1 gp120, can arise from infectious viral particles, defective virions, or freely circulating soluble proteins that have been shed from virions. Tissue concentrations are disproportionately higher than plasma levels, persist even when patients are on effective ART, and may be underestimated by current techniques used to measure them [27], [28], [29]. The expression of gp120 in liver tissue has not been previously reported.

The aims of this study were to determine the effect of X4 gp120 on HSC activation and collagen I expression through interactions with CXCR4, explore underlying intracellular signaling pathways, and determine whether gp120 is present in liver tissue from HIV/HCV coinfected patients prior to initiation of anti-retroviral therapy.

## Materials and Methods

### Reagents

Recombinant HIV-1 HXB gp120 was kindly provided by Dr. Robert Doms (Univ of Pennsylvania, Philadelphia, PA) and AT-2 treated HIV-IIIB was provided by Dr. J. Lifson through the NIH AIDS Research and Reference Reagent Program, Division of AIDS, NIAID. Phosphorylation-specific polyclonal antibodies against extracellular signal-regulated kinase (ERK) and Akt (Ser 473) were purchased from Cell Signaling Technology (Beverly, Massachusetts, USA). The ERK inhibitor UO126 was purchased from Merck Chemicals (Nottingham,UK).

### Isolation and culture of primary human HSCs

Primary HSCs were isolated from normal liver margins in patients undergoing hepatic resection for primary benign tumors or a single metastasis from colon cancer, as previously described [24]. Briefly, immediately post-hepatectomy, an isolated liver section was washed and the portal vessels cannulated for *in-situ* digestion with pronase (Roche Applied Sciences GmbH, Mannheim, Germany) and collagenase B (Roche Applied Sciences), followed by density gradient centrifugation. HSC purity was consistently found to be between 95–99% with a viability of 95%. HSCs were plated on plastic in Dulbecco's Modified Eagle Medium (DMEM) (Invitrogen, Carlsbad, CA) supplemented with 10% Fetal Bovine Serum (FBS) in a 5% CO_2_ humidified atmosphere. HSCs were activated by culturing on plastic for 7–12 days and subcultured to passage 3 for all experiments.

### Cells lines

LX-2 cells, an immortalized human hepatic stellate cell line, were cultured and characterized as previously described [30]. For the studies focused on determining the impact of HIV X4 gp120 protein on the activation and expression of collagen I, cells were serum starved for 12–24 hours prior to X4 gp120 challenge. To confirm that gp120 effects were CD4-independent, HSCs were pre-incubated with 25 µg/mL anti-CD4 (BD Biosciences, clone Leu 3A) for 30 minutes prior to challenge with X4 gp120.

### Reverse-Transcription and Real-time quantitative PCR (qRT-PCR)

RNA was extracted from the cells and reverse transcribed into cDNA using RNeasy® kit (Qiagen, Valencia, CA) and Omniscript RT Kit (Qiagen), respectively. mRNA levels were analyzed by qRT-PCR using SYBR green qRT-PCR Master Mix (Roche) on the LightCycler®480 System (Roche). Primer sequences are listed in Table S1.

### Knockdown of CXCR4 in Hepatic Stellate Cells

CXCR4 knockdown was accomplished using short hairpin RNA (shRNA). Primary human HSCs were grown in 6-well culture plates to 60–70% confluence. Cells were transfected with either shCXCR4 (Sigma, St. Louis, Catalog #SH0221) (or a shRNA control vector (pLKO.1, Sigma Catalog # SHC001) using TransIT-LT1 reagent (Mirus Bio, Madison, WI). After 6 hours, the medium was replaced with normal growth medium for 72 hours which leads to maximal knockdown of CXCR4 [24].

### Immunoblots

Cell extracts were prepared by pelleting the cells with lysis buffer (Roche Applied Sciences) containing protease and phosphatase inhibitors. (Roche Applied Sciences and Thermo Scientific). Protein concentration were determined with the Bradford method using Bio-Rad DC protein assay kit (Bio-Rad). Equal amounts of protein were separated by SDS-PAGE, transferred to Hybond C membranes, and immunoblotted with specific primary antibodies for collagen I (Rockland Immunochemicals, Inc., Gilbertsville, PA), a-SMA (Promega), and ß-actin (Sigma), and visualized using a secondary antibody conjugated to horseradish peroxidase (HRP) (GE Healthcare) and revealed with a Western chemiluminescent HRP substrate detection system (Millipore Billerica, MA).

### Immunohistochemistry

For gp120 staining, deidentified, formalin-fixed, paraffin-embedded liver biopsies were obtained from HIV/HCV coinfected patients as part of their standard of care and provided by the Mount Sinai Department of Pathology with the approval of Mount Sinai IRB (GCO# 05-1343). 5 µm tissue sections were sequentially deparaffinized followed by autoclaving for epitope retrieval. Tissues were then blocked for 1 hr in 5% BSA/PBS at room temperature and incubated in primary antibody overnight at 4°C with a 1∶100 dilution of mouse monoclonal anti-gp120 (Advanced Bioscience Laboratories, Inc., Kensington MD or Abcam, Cambridge, MA). This antibody cross-reacts with the gp120 of a number of both X4 and R5-tropic HIV-1 isolates. Sections were revealed using DAKO EnVision HRP-DAB kit and counterstained with hematoxylin. For negative staining controls, the tissue sections were incubated with isotype control antibody.

### Statistical Analysis

All results are expressed as the mean +/− standard deviation. Statistical significance was tested using unpaired Student t test, and P<0.05 was considered significant.

### Ethics Statement

For primary cell isolation and immunohistochemistry, deidentified waste or archived tissue, respectively, was provided to the investigators. The Mount Sinai IRB exempted this study from review (Exempt Category #4) because samples were considered waste or archived material and waived the need for consent due to the fact that the samples received were deidentified and the investigators had no way of tracking the samples back to the individual patient.

## Results

### X4 HIV-1 gp120 promotes expression of fibrogenic markers in activated human hepatic stellate cells

**Figure 1:**
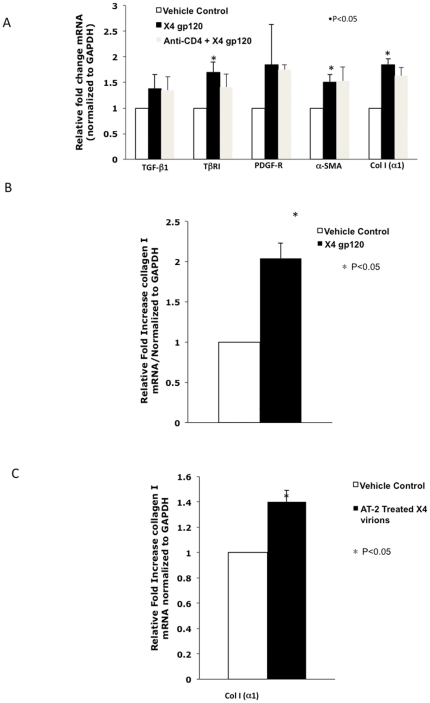
X4 HIV-1 gp120 induces fibrogenic gene expression in human stellate cells. (A) LX-2 cells were serum-starved for 24 hrs, treated with X4 HIV-1 gp120 at a final concentration of 500 ng/ml for 2 hours, RNA harvested, reverse transcribed, and qRT-PCR performed for TGF-ß1, type I TGF-ß receptor, a-SMA and coll I (a1) mRNA levels. To confirm X4 gp120 effects were CD4-independent, cells were pre-incubated with 25 µg/mL anti-CD4 30 minutes prior to challenge with X4 gp120. (B, C) Effect on collagen I (a1) was confirmed in primary HSCs with both X4 gp120 as well as AT-2 treated X4-tropic HIV-IIIB, which presents gp120 in its oligomeric confirmation. All data are expressed as means +/− standard deviation of at least three independent experiments.

The HIV envelope protein, gp120, has been shown to promote hepatocyte apoptosis, hepatocellular secretion of the pro-inflammatory cytokine IL-8 [17], elicit pro-inflammatory and to a lesser extent pro-fibrogenic effects on HSCs, and promote the directional migration of hepatic HSCs [26]. As expression of CXCR4 increases with HSC activation, we examined the pro-fibrogenic effects of X4 tropic gp120 on activated HSCs. LX-2 cells were treated with X4 gp120 (HXB) and fibrogenic markers examined by qRT-PCR. As shown in Figure 1A, X4 gp120 resulted in a significant increase in collagen I, a-SMA, and type I TGF-ß receptor (TßRI) expression after 2 hours which was not blocked by pre-incubation with anti-CD4 antibody. The effect of X4 gp120 on collagen I a1 mRNA levels was further confirmed in passage #3 primary HSCs (Figure 1B). In addition, AT-2 treated HIV IIIB (X4-tropic), which maintains the gp120 in its natural oligomeric conformation, also resulted in a significant increase in collagen I a1 mRNA expression (Fig. 1C). These results suggest direct pro-fibrogenic effects of X4 gp120 on HSCs.

### X4 HIV-1 gp120 promotes a-SMA and collagen I expression in activated Human Stellate Cells

**Figure 2:**
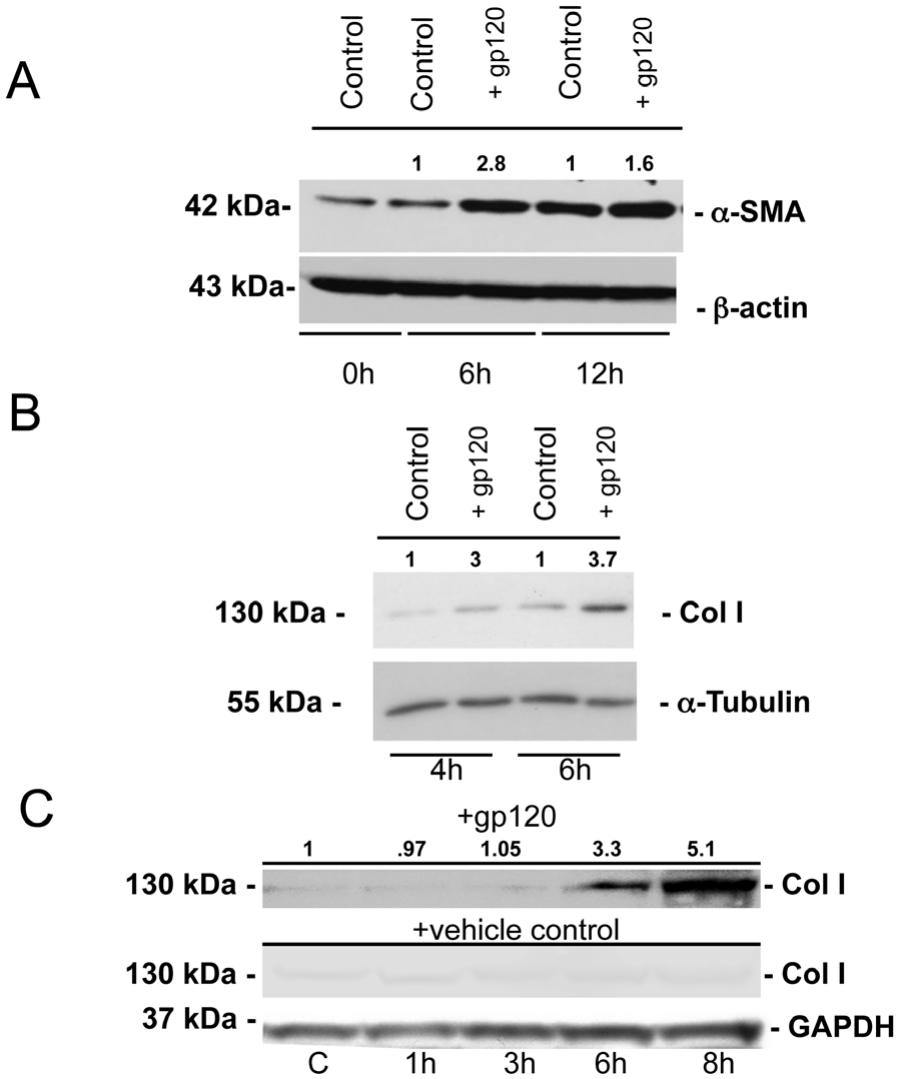
X4 HIV-1 gp120 promotes a-SMA and collagen I protein expression in human HSCs. (A, B) LX-2 cells were incubated with 500 ng/ml X4 gp120 for 0–12 hours, protein harvested, and expression of a-SMA and collagen I examined by Western blot analysis. (C) The effect of X4 gp120 versus vehicle control on collagen I protein expression was further confirmed in passage #3 primary HSCs. Densitometry was performed and normalized arbitrary units represented numerically.

The protein expression of both a-SMA, a marker of activated HSCs, and collagen I was further assessed by Western blot. Both LX-2 and primary human HSCs demonstrated increased a-SMA and collagen I protein expression peaking at 6–8 hours after gp120 treatment compared with control (Fig. 2 A–C). These results confirm the direct pro-fibrogenic effects of X4 gp120 on HSCs.

### X4 gp120 induction of a-SMA and collagen I expression is CXCR4-dependent

**Figure 3:**
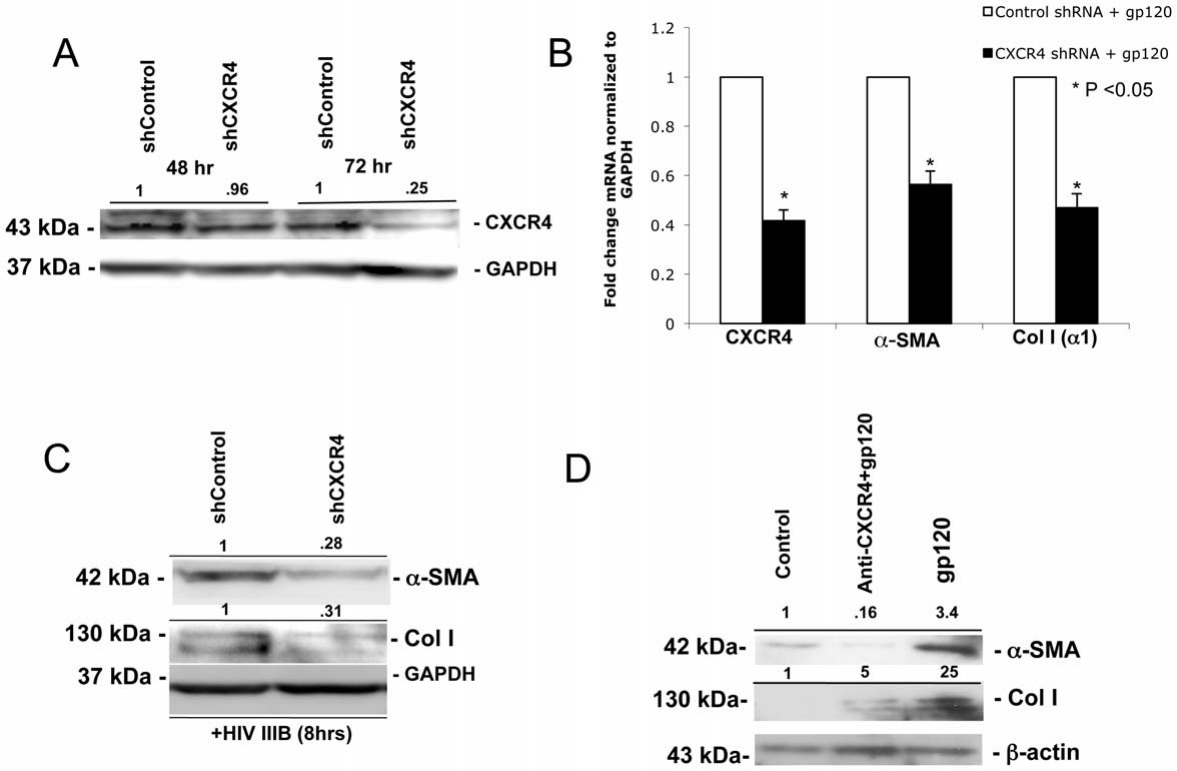
X4 gp120 induction of a-SMA and collagen I expression is CXCR4-dependent. (A) Passage #3 primary HSCs were plated at a density of 2×10^4^ cells/well in 6-well plates, serum-starved for 24 hours, and then transfected with shCXCR4 or shControl. A 75% reduction in CXCR4 protein expression was noted 72 hours after transfection by Western blot. (B) 72 hours after shControl or sh CXCR4 transfection, primary HSCs were then challenged with X4 gp120 for 2 hours, RNA harvested, reverse transcribed and qRT-PCR performed for CXCR4, a-SMA, and coll I (a1). A 40–50% reduction in gp120-induced collagen (a1) and a-SMA mRNA levels was observed with CXCR4 knockdown. Data are expressed as the mean ± standard deviation of three independent experiments. (C) 72 hours after shCXCR4 knockdown, HSCs were challenged with HIV-IIIB for 8 hours and cell lysates used for Western blot where a 70% reduction in the protein expression of both a-SMA and collagen I was observed. (D) Human primary HSCs were pretreated with anti-CXCR4 antibody for 30 min (20 µg/ml) followed by treatment with gp120 (500 ng/mL) for 8 hours. Both X4 gp120-induced a-SMA and coll I protein expression were attenuated by anti-CXCR4 neutralizing antibody. ß-actin, a-tubulin, and GAPDH were used as loading controls. Representative Western blots with normalized densitometric arbitary units shown.

To determine whether gp120 induction of a-SMA and collagen I were CXCR4-dependent, two approaches were utilized. First, CXCR4 was knocked down using CXCR4-targeting shRNA (shCXCR4) vs. non-targeting shRNA (shControl) followed by treatment with X4 gp120. We have previously shown that peak reduction in CXCR4 protein expression occurs 72 hours after transfection [24], which was again confirmed for experiments outlined here (Figure 3A). Therefore, cells were treated with X4 gp120 or HIV IIIB 72 hours after treatment with shCXCR4. By qRT-PCR a greater than 40% reduction in a-SMA and collagen I mRNA was observed while a 70% reduction in a-SMA and collagen I protein levels were seen by Western blot in the presence of shCXCR4 (Fig. 3B,C). Furthermore, preincubation of human primary HSCs with anti-CXCR4 neutralizing antibody for 30 min prior to X4 gp120 treatment resulted in an attenuation of X4 gp120-mediated a-SMA and collagen I induction (Fig. 3D). Taken together, these results indicate that X4 gp120 induction of a-SMA and collagen I expression in HSCs occurs in a CXCR4-dependent fashion.

### X4 gp120 induction of collagen I occurs via the ERK 1/2 pathway

**Figure 4:**
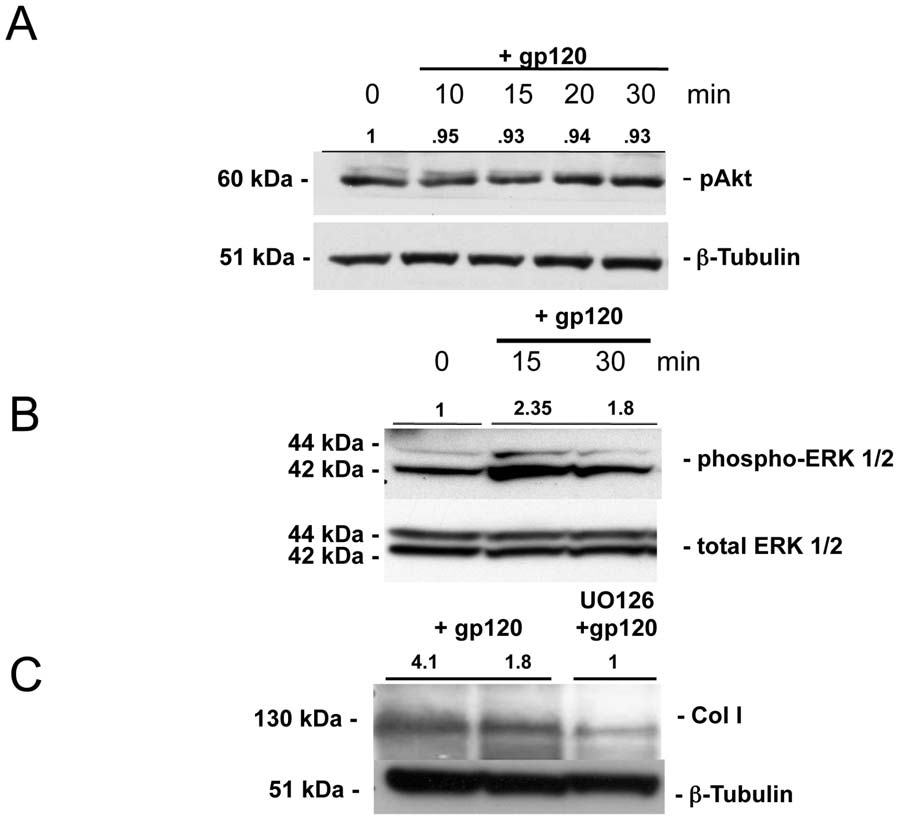
X4 gp120 induction of collagen I occurs via the ERK 1/2 pathway. (A, B) To determine whether X4 gp120 activates PI3K-Akt and/or ERK 1/2 pathways, primary HSCs were incubated with 500 ng/ml of X4 gp120 and levels of phospho-Akt and phospho-ERK 1/2 assessed by Western blot. An increase in phospho-ERK 1/2 was seen while no increase in phospho-Akt was seen in response to X4 gp120. (C) Pretreatment of cells with ERK inhibitor (UO126; 10 nM) 30 minutes prior to gp120 treatment resulted in a marked decrease in X4 gp120-induced collagen I protein expression in HSCs. ß-tubulin and total ERK1/2 were used as loading controls. Representative Western blots of three independent experiments with normalized densitometric units shown.

We next explored the intracellular signaling pathways responsible for the biological effects of X4 gp120 on collagen I expression in HSCs. Both ERK 1/2 and phosphatidylinositol 3-kinase-Akt (PI3K-Akt) pathways are important for mediating effects of SDF-1a on collagen expression in HSCs via CXCR4 [24]. We therefore examined whether X4 gp120 induces phosphorylation of ERK 1/2 and Akt in primary HSCs by Western blot. Treatment with gp120 induced phosphorylation of ERK 1/2 but not Akt (Fig. 4 A, B). Moreover, pretreatment with the ERK inhibitor (UO126; 10 nM for 30 min) significantly decreased X4 gp120-induced collagen I in HSCs (Fig. 4C). These data implicate the ERK 1/2 pathway in promoting X4 gp120 induced collagen expression in HSCs.

### Gp120 is present in liver tissue from HIV/HCV infected patients

**Figure 5:**
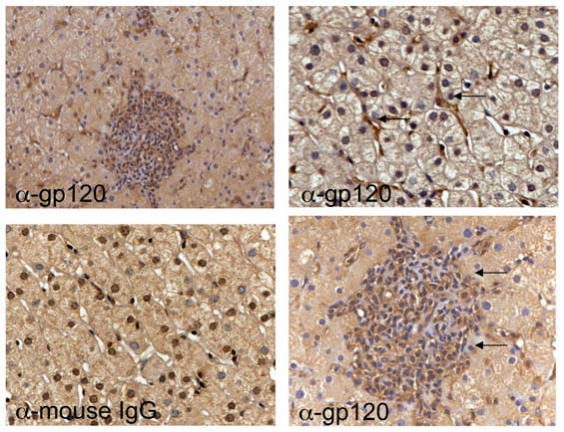
Gp120 is present in liver tissue from HIV/HCV infected patients. Paraffin-embedded liver biopsies from HIV/HCV coinfected patients prior to initiation of ART were immunostained with monoclonal anti-gp120 or isotype control. Sinusoidal staining was appreciated in all 3 specimens. Representative images shown (Magnification 100×, 630×). Viral load ranges: HIV RNA levels ranged from 514–2,455 copies/ml; HCV RNA levels ranged from 1.2 million–1.6 million IU/ml.

Numerous studies have examined the effect of gp120 on intrahepatic cell populations, but the expression of gp120 in human liver samples has not been reported. We therefore examined the expression of gp120 in liver biopsies from 3 HIV/HCV coinfected patients prior to the initiation of combined anti-retroviral therapy. Immunohistochemistry for gp120 on paraffin-embedded liver biopsies from HIV/HCV coinfected patients demonstrated sinusoidal staining in all 3 patients (Fig. 5). While the cellular source of the gp120 requires further investigation, gp120 is present in the sinusoid where stellate cells reside as well as in areas of portal inflammation.

## Discussion

We have previously established that activated HSCs express CXCR4 in HCV-infected livers [24], culture-induced HSC activation leads to increased CXCR4 expression [24], and the endogenous ligand for CXCR4, SDF-1a, elicits pro-fibrogenic effects in activated HSCs. We have also demonstrated that HIV infection of HSCs is associated with increased collagen I expression but in the current study we show that X4 gp120, the envelope protein of X4-tropic HIV-1, can promote HSC activation and collagen I expression in HSCs even in the absence of infection through its interactions with CXCR4. While other *in vitro* studies have utilized gp120 to examine effects on intrahepatic cell populations, we show for the first time that gp120 is present in the livers of patients with HIV/HCV coinfection. Since the number of cells infected by HIV *in vivo* is low compared to circulating and tissue concentrations of viral proteins and non-infectious virion particles exist in excess of infectious virus, the interaction between HIV gp120 and HSCs may be highly relevant and targetable.

It has been reported that R5 gp120 promotes the secretion of IL-6 and MCP-1 by HSCs, promotes the chemotaxis of HSCs, and to a lesser extent exerts some pro-fibrogenic effects. Its chemotactic effects were found to be mediated through PI3K/Akt pathways while its pro-inflammatory effects were mediated through NF-kB and p38^MAPK^ [26]. According to this report, IIIB (X4-tropic) gp120 induced HSC migration less effectively than R5 tropic gp120 and therefore the authors focused more on R5 gp120 effects. R5-tropic virus predominates early in infection while X4-tropic HIV emerges later in the course of disease and is associated with rapid HIV disease progression. In this study, we focused on the effect of X4-tropic gp120 and found that the ERK 1/2 pathway was important for collagen I induction while activation of the PI3K/Akt pathway was not observed. It is possible that R5 gp120 exerts more pro-inflammatory effects while X4 gp120 is more pro-fibrogenic and requires further investigation. Interestingly, the endogenous ligand for CXCR4, SDF-1a, induced collagen I expression in activated HSCs through both ERK 1/2 and PI3K/Akt pathways [24]. While X4 gp120 and SDF-1a can activate similar pathways, they can activate different pathways depending on cell type and context [31], [32]. Given the significant impact of gp120 on the protein expression of a-SMA and collagen I and the modest increase in mRNA levels, it is likely that post-transcriptional factors are predominantly involved. While classically the ERK1/2 pathway regulates genes at the transcriptional level, over 180 targets have been identified including cytoskeletal proteins, signaling proteins, etc [33]. Moreover, while collagen I production by activated hepatic stellate cells is partially regulated at the transcriptional level, there is increasing evidence for translational regulation. For example, leucine upregulates collagen I production in hepatic stellate cells at the translational level and involves the ERK-1/2 pathway [34]. Future studies will need to dissect the exact mechanism by which gp120 upregulates collagen I expression via the ERK1/2 pathway.

One critical issue is whether concentrations used to elicit biologic effects *in vitro* are present *in vivo*. Because of the presence of anti-gp120 abs, the effective amount of gp120 available for binding to receptor in the plasma of HIV infected patients is very low [35]. However, tissue concentrations are disproportionately higher than plasma levels, persist even when patients are on effective ART, and may be underestimated by current techniques used to measure them [27], [28], [29]. The hepatic concentration of gp120 in HIV patients is not known, yet many studies have examined the *in vitro* effects of gp120 on hepatic cell types [16], [18], [36]. In the current study, we demonstrate for the first time the presence of gp120 both in areas of portal inflammation as well as in the sinusoidal space of livers in patients with HIV/HCV coinfection. Since this antibody cross-reacts with the gp120 of a number of X4 and R5-tropic isolates, it is not clear whether X4, R5, or both are present in these particular samples and future studies using carefully phenotyped patient samples will be needed to specifically determine whether fibrogenic effects are more attributable to X4 or R5 gp120. The potential cellular sources of gp120 include Kupffer cells, CD4 cells, sinusoidal endothelial cells, and stellate cells; though the most likely candidate are Kupffer cells since they are the only hepatic cell type clearly shown to be infected by HIV *in vivo* and *in vitro* [*37]*, [38**]. Nevertheless, the presence of gp120 in the sinusoid suggests that gp120 interactions with CXCR4 or CCR5 on HSCs is likely. In addition, future studies with a large number of patient samples will allow us to quantify gp120 levels and correlate with HSC activation and fibrosis in patients with HIV/HCV and HIV monoinfection. We postulate that any underlying chronic liver injury results in stellate cell activation and increased CXCR4 and CCR5 expression. Due to shared routes of transmission, HCV is the most common co-factor but toxicity from ARVs, alcohol, and steatosis are additional factors important factors in promoting stellate cell activation in HIV monoinfected patients. Therefore, HIV gp120 may further enhance HSC activation and ECM production accelerating progression of liver fibrosis induced by HCV and other forms of chronic liver injury. In two recent studies [39], [40], HIV viremia was associated with liver fibrosis in HIV monoinfected patients highlighting the potential importance of HIV interactions with hepatic stellate cells even in the absence of a hepatotrophic virus and therefore the importance of targeting this interaction therapeutically. Our findings add support to the early initiation of ARVs in patients with any underlying chronic liver injury or specific targeting of the gp120-chemokine co-receptor interaction.

In addition to direct effects of X4 gp120 in upregulating collagen I expression in HSCs, we found that X4 gp120 significantly increased TßRI expression and a trend toward increased TGF-ß1 expression by qRT-PCR was also observed. TGF-ß1 is the most profibrogenic cytokine in chronic liver disease and is elevated in the serum of HIV-infected patients [41]. A trend toward increased intrahepatic expression of TGF-ß1 in HIV/HCV coinfected livers was also reported [42]. As others have reported that both X4 and R5 gp120 induce TGF-ß1 by HCV replicon cells [18], these findings suggest that X4 gp120 may promote fibrosis both directly and via induction of TGF-ß1 by both parenchymal and non-parenchymal cells of the liver.

In summary, we demonstrate that gp120 is present in HIV/HCV coinfected livers and X4 gp120 elicits pro-fibrogenic effects on activated HSCs via a direct interaction with CXCR4. The availability of small molecule inhibitors to CXCR4 make this a potential anti-fibrotic target in HIV/HCV coinfected patients.

## Supporting Information

Table S1 **Human Primer Sequences for qRT-PCR.** RNA extracted from LX2 cells and primary HSCs after gp120 challenge, reverse transcribed and mRNA levels of TGF-ß1, type I TGF-ß receptor, a-SMA and coll I (a1) mRNA levels assessed by real-time PCR using human primer sequences listed.
(TIFF)
