# Supplementary figures and images for: X4 Human Immunodeficiency Virus Type 1 gp120 Promotes Human Hepatic Stellate Cell Activation and Collagen I Expression through Interactions with CXCR4
Source: PLoS One. 2012 Mar 27;7(3):e33659. doi: 10.1371/journal.pone.0033659 (PMC3313947; doi:10.1371/journal.pone.0033659)

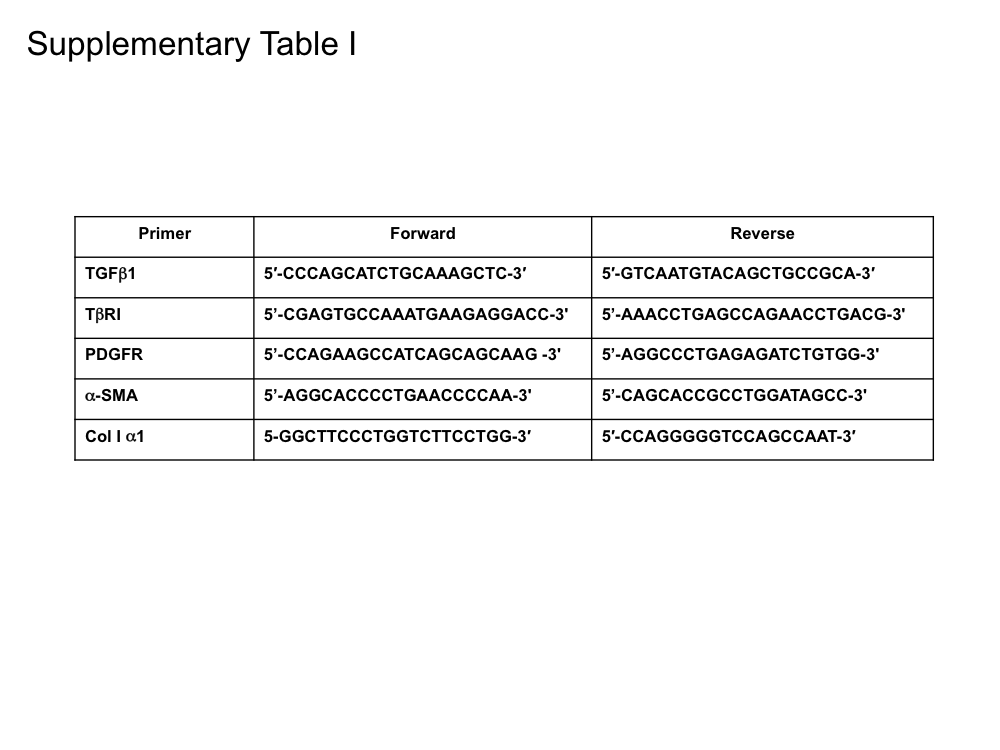

Supplement: Table S1 — Human Primer Sequences for qRT-PCR. RNA extracted from LX2 cells and primary HSCs after gp120 challenge, reverse transcribed and mRNA levels of TGF-ß1, type I TGF-ß receptor, a-SMA and coll I (a1) mRNA levels assessed by real-time PCR using human primer sequences listed. (TIFF) [file pone.0033659.s001.tiff]
